# *Bacillus velezensis* S141: A Soybean Growth-Promoting Rhizosphere Bacterium

**DOI:** 10.3390/plants15030387

**Published:** 2026-01-27

**Authors:** Ken-ichi Yoshida, Neung Teaumroong

**Affiliations:** 1 Department of Science, Technology and Innovation, Kobe University, 1-1 Rokkodai, Nada, Kobe 657-8501, Japan; 2 Institute of Agricultural Technology, Suranaree University of Technology, 111 University Avenue, Suranaree Sub-District, Muang District, Nakhon Ratchasima 30000, Thailand; neung@sut.ac.th

**Keywords:** *Bacillus velezensis*, *Bradyrhizobium diazoefficiens*, nodulation, soybean

## Abstract

Soybean (*Glycine max*) is a globally important crop, as it has high protein and lipid content and plays a central role in sustainable agriculture. Recent advances in rhizosphere biology have highlighted the critical role of soybean root exudates, particularly isoflavones and other secondary metabolites, in shaping microbial community structure and function. These exudates mediate complex, bidirectional signalling with rhizosphere microorganisms, influencing nutrient acquisition, stress resilience, and disease suppression. This review describes current knowledge on soybean–microbe interactions, with a focus on the emerging concept of the rhizosphere as a dynamic communication network. Particular attention is given to *Bacillus velezensis* S141, a plant growth-promoting rhizobacterium (PGPR) with distinctive traits, including β-glucosidase-mediated isoflavone hydrolysis, phytohormone production, and drought resilience. Coinoculation studies with *Bradyrhizobium* spp. demonstrate enhanced nodulation, nitrogen fixation, and yield, supported by transcriptomic and ultrastructural evidence. Comparative genomic analyses further underscore host-adaptive features of S141, distinguishing it from other *Bacillus* strains. Despite promising findings, mechanistic gaps remain regarding metabolite-mediated signalling and environmental robustness. Future research integrating metabolomics, synthetic ecology, and microbial consortia design will be essential to harness rhizosphere signalling for climate-resilient, low-input soybean cultivation.

## 1. Introduction

Global production of soybeans (*Glycine max*) exceeds 400 million tonnes annually, with Brazil and the United States as the leading producers [1,2]. Soybeans are particularly valued for their nutritional quality, as the seeds are rich in protein and lipids, distinguishing them from other staple crops such as corn (*Zea mays*), rice (*Oryza sativa*), and wheat (*Triticum aestivum*) [3]. In Japan, soybeans serve as an essential raw material for traditional foods such as tofu, natto, soy sauce, and miso, whereas in many other regions they are primarily processed into oil and animal feed.

Soybeans produce unique secondary metabolites, such as isoflavones and saponins, and secrete them into the rhizosphere [4,5]. The rhizosphere, defined as ‘the area of soil affected by roots’ [6], is a zone where metabolites are actively secreted from roots [7,8] and additionally released when root tissues slough off from the main root axis [9]. Microorganisms inhabiting the rhizosphere are strongly influenced by these exudates [10,11,12,13]. For example, rhizobia and arbuscular mycorrhizal fungi establish symbiotic associations with soybean roots through secretion-mediated interactions that enhance plant growth. These microorganisms provide soybeans with nitrogen and phosphorus, respectively, suggesting that chemical fertilisers should, in theory, be unnecessary for cultivation. In practice, however, large quantities of fertilisers are often applied to maximise yield, which contributes to environmental problems such as eutrophication of rivers and lakes [14]. Consequently, the strategic utilisation of rhizosphere microorganisms represents a key avenue for achieving sustainable soybean production [15].

Isoflavones and strigolactones secreted from soybean roots act as signalling molecules in symbiosis with rhizobia and arbuscular mycorrhizal fungi, respectively [7]. In addition, glyceollin is secreted as a disease-inducible phytoalexin, while glycinoeclepin A functions in promoting the hatching of soybean cyst nematodes [16]. The structure and activity of microbial communities in the rhizosphere differ markedly from those in bulk soil. In general, microbial diversity tends to decline in proximity to plant roots [17,18,19,20]. This observation has led to the suggestion that plants exert selective control over microorganisms in the rhizosphere [21,22,23,24]. Moreover, recent metagenomic and community synthesis studies have identified additional factors such as microbial interactions, host immunity, and the dynamics of exudate chemistry [25].

Extensive studies show that climate, soil type, plant species, plant genotype, and developmental stage are key factors shaping the diversity and composition of rhizosphere microbial communities [26,27,28]. Conversely, the rhizosphere microbiome influences plant performance, affecting nutrient acquisition, disease suppression, and resistance to both biotic and abiotic stresses [29,30,31,32]. Numerous studies have investigated the soybean rhizosphere microbiome (including bacteria and fungi), demonstrating that the soybean rhizosphere harbours significantly more symbiotic rhizobia than the surrounding soil [33,34,35,36,37].

Fertilisation and cropping systems modulate the rhizosphere signalling environment, as repeatedly highlighted in recent meta-analyses and reviews. In particular, it has been argued that sugar secretion in the rhizosphere acts as a “driver” determining the selective composition of microbial communities [38]. Whilst fertilisation significantly alters soil chemistry, microbial community diversity often remains relatively stable. This suggests that fertilisation acts as an “external control input” that rewires the importance of specific nodes or modules within the network.

During the growing season in soybean fields, the bacterial community in the rhizosphere changes, while the overall soil composition remains nearly unchanged [37]. Therefore, rhizosphere bacterial dynamics are driven primarily by plant growth rather than environmental variation. Plant growth-promoting rhizobacteria (PGPR), including *Bradyrhizobium*, *Bacillus*, and related taxa, occur in higher abundance in rhizosphere soil than in bulk soil. In soybean fields, *Bradyrhizobium diazoefficiens* and *Bradyrhizobium elkanii* are the predominant species forming root nodules responsible for nitrogen fixation [39]. In a separate study, although distinguishing *Bradyrhizobium* species or strains at the field scale was limited by sequencing resolution, members of the genus displayed distinct responses at the operational taxonomic unit level [40].

The fungal community in the rhizosphere, by contrast, remained relatively stable at the phylum level throughout soybean growth, with Ascomycota and Basidiomycota showing the greatest abundance [41]. However, analyses based on internal transcribed spacer regions revealed that the growth stage of soybean strongly influences fungal community diversity [42]. Moreover, fungal composition is also modulated by fertiliser application and rhizobium inoculation [42,43].

Research on black soybean fields suggested that the bacterial community in the rhizosphere influences soybean production [44]. The yield of black soybeans cultivated in the mountainous areas surrounding central Kyoto declined without obvious pathogen symptoms, prompting an investigation into the involvement of the microbial community [45]. Variations in soil bacterial communities at farms under similar climatic conditions indicated the impact of management practices on these assemblages. The rhizosphere communities of each farm differed significantly from the bulk soil, with *Bradyrhizobium* and *Bacillus* being the dominant taxa. Network analysis using the Confeito algorithm further suggested a link between rhizosphere bacterial structure and soybean growth [44]. (The Confeito algorithm is a size-sensitive community detection method for visualising and analysing the network structure of microbial communities, excelling particularly in extracting small-scale, functional modules within correlation networks [46,47].)

In vitro experiments have been conducted to investigate the impact of root-derived metabolites on microbial communities [48,49,50,51,52,53,54,55,56,57,58,59]. Although the use of root exudates in artificial soil systems is limited by in vitro methodology, it suggests that compounds secreted by roots may influence rhizosphere microorganisms. The effects of root exudates from three generations of thale cress (*Arabidopsis thaliana*) and alfalfa (*Medicago truncatula*) on fungal communities in native soil were both qualitatively and quantitatively comparable to those of living plants [48]. Root exudates from *A*. *thaliana* were fractionated into natural phytochemical mixtures and subsequently applied to soil. Phenolic compounds showed a positive correlation with the abundance of bacteria [49]. Similarly, the flavonoid 7,4′-dihydroxyflavone, obtained from alfalfa root exudates, functions as an inducer of *nod* genes required for nod factor biosynthesis, thereby influencing interactions with diverse soil bacteria when added to soil [50]. The association between root-secreted metabolites and microbial communities has also been observed during plant growth, suggesting that exudates may play a regulatory role in shaping rhizosphere communities [26,51]. Comparative genomics and exometabolomics analyses of slender wild oat *Avena barbata* further support the idea that aromatic organic acids secreted from roots are a significant factor contributing to the assembly of rhizosphere microbial communities [52]. Research on the metabolites exuded from soybean roots has focused on their interactions with plant PGPR and the degradation of polycyclic aromatic hydrocarbons (PAHs), significant environmental pollutants [53,54]. Inoculation with *Pseudomonas oryzihabitans* altered the composition of soybean root exudate, resulting in reduced levels of sugars and amino acids [53]. Moreover, the addition of soybean root exudates to PAH-contaminated soil significantly enhanced PAH degradation by soil bacteria [54]. In a 13-year soybean monoculture experiment, the concentrations of the isoflavones daidzein and genistein in the rhizosphere correlated with changes in the soil microbial community, with genistein in particular linked to the mycelial growth of arbuscular mycorrhizal fungi [55,56]. The connection between flavonoid secretion and rhizobial interactions is further supported by the identification of quantitative trait loci that affect both rhizobial affinity and the release of genistein [57]. In addition, analysis of rhizosphere bacterial communities in root hairs of plants where isoflavone synthase was silenced revealed that isoflavones exert a modest but significant effect on bacterial composition, especially impacting the families Comamonadaceae and Xanthomonadaceae [58].

As already mentioned, *Bradyrhizobium* species play a key role in the nitrogen cycle of the agroecosystem by infecting soybean roots and forming nitrogen-fixing nodules, where a substantial amount of nitrogen is fixed and transferred to the plant, thereby reducing the need for nitrogen fertilisers [39]. However, the establishment of nitrogen-fixing symbiosis is inhibited by several factors. Lack of nodules or ineffective nodulation in soybean roots has been attributed to incompatibility between the symbiotic bacteria and the host, as well as the influence of known and unknown biomolecules such as flavonoids, polysaccharides, and hormones [59]. *Bradyrhizobium* inoculum levels are also an important factor in the agricultural application of microbial inoculants, and many countries have established standards for them [60]. Field experiments required inoculating bacterial densities between 10^3^ and 10^6^ colony-forming units (CFU)/mL per seed; however, higher inoculum doses did not lead to an increase in nodule numbers [61]. These results suggest notable implications; however, we cannot draw definitive conclusions since the threshold for microbial saturation in root establishment remains unclear.

In recent years, rhizosphere signalling has been reinterpreted from a traditional understanding centred on molecular pathways as a multi-layered network woven by plants, microorganisms, and environmental factors. Particular emphasis has been placed on the role of root exudates as a “field” that induces the aggregation and functional differentiation of microbial communities [62]. Evidence indicates that compositional shifts in primary and secondary metabolites influence microbial community topology and functional module formation, clearly demonstrating that rhizosphere signalling is intrinsically linked to community-level phenomena. Furthermore, plant–microbe interactions in the rhizosphere are organised not as a unidirectional release of plant-derived signals, but as bidirectional communication networks involving microbial metabolites [63,64]. For instance, consider an indole-3-acetic acid (IAA) analogue produced by rhizobacteria. This metabolite interacts with plant roots, influencing auxin pathways and resulting in altered root architecture. As the roots grow in response to these microbial signals, the changed root structure can affect the release of exudates, thereby feeding back into the microbial community. Such interactions vividly illustrate the concept of multipoint communication, where microbial metabolites and root responses are part of a continuous dialogue. Hormone analogues, volatile organic compounds, siderophores and other substances produced by microbes alter local root morphogenesis and nutrient acquisition strategies, thereby reorganising nutrient gradients and microbial network structures within the rhizosphere. These findings indicate that rhizosphere signalling should be understood as multipoint communication. The proposed “probiotic model” provides a framework for analysing interactions between root exudates and functional microbial communities under controlled conditions, demonstrating that carbohydrate-rich mucilage drives the enrichment of nitrogen-fixing bacteria and the formation of pathogen suppression networks [65]. This model introduces a control theory perspective, treating the rhizosphere as an “input (secretions)—network (microbial interactions)—output (nutrient acquisition/disease suppression)” system, thereby extending the conceptual foundation for signal transduction research.

Promoting soybean nitrogen fixation by inoculating PGPR together with *Bradyrhizobium* species as bidirectional communication networks for symbiotic nitrogen fixation is an attractive strategy to enhance sustainable agricultural production systems [66,67,68,69,70,71,72,73]. For example, precedents exist for *Azospirillum* [66], *Azotobacter* [67], *Bacillus* [68], *Serratia* [69], and *Streptomyces* species [70]. Various PGPR coexist with *Bradyrhizobium* species in rhizosphere soils and interact during root colonisation. Compared with *Bradyrhizobium* inoculation alone, co-culture with *Azospirillum* can increase the number of root hairs, flavonoid exudation from roots, and the number of nodules formed [71]. The beneficial effects of PGPR are thought to arise from their ability to produce phytohormones [72] and from their capacity to stimulate the production and secretion of other metabolites, such as siderophores and flavonoids, that promote the expression of the genes for nodule formation [73]. However, it is unlikely that any single rhizobacterial strain will dominate and perform effectively across different environments; therefore, mixtures of compatible strains may be more effective in promoting plant growth than individual species.

As revealed by these findings, some microbial species preferentially colonise the rhizosphere, establishing active symbiotic relationships. The studies further suggest the presence of additional non-symbiotic microorganisms that contribute to plant growth, and efforts are ongoing to identify them from multiple perspectives. One microorganism isolated as a non-symbiotic microorganism that significantly promotes soybean growth is *Bacillus velezensis* S141. This bacterial strain promotes soybean growth through several biotic interactions, both within plant-associated microbial communities and among different microbial species. Its mechanisms include hormone synthesis, isoflavone hydrolysis, enhanced nitrogen fixation, and improved drought tolerance. These features make it a compelling subject that extends beyond traditional views of PGPR. This review summarises research on the role of this microorganism and presents the current status and prospects.

## 2. *B. velezensis* S141 Was Isolated as a PGPR from a Soybean Field in Thailand

In the quest to enhance symbiotic nitrogen fixation in soybean (*Glycine max*), *Bradyrhizobium diazoefficiens* USDA110 and THA6 were coinoculated with 285 bacterial isolates collected from soybean rhizosphere soils across Thailand [74]. Of these, 12 isolates turned out to enhance nodulation and plant biomass. Finally, strain S141 consistently showed the most pronounced effects when coinoculated with both USDA110 and THA6.

The soybean gene *Glyma17g07330*, involved in nodulation signalling, and bacterial genes such as *nifH* (nitrogenase reductase), *phbC* (polyhydroxybutyrate synthesis), *dctA* (C4-dicarboxylate transporter), and *otsA* (trehalose synthesis) are key regulators of nitrogen fixation, carbon metabolism, and stress tolerance, and expression of these genes in soybean nodules was analysed at multiple stages post-inoculation to investigate the molecular basis of the impact of S141 [74]. Coinoculation with S141 notably influenced *nifH*, which peaked at four weeks and remained elevated at seven weeks, indicating sustained nitrogenase activity. The expression of *phbC*, *dctA*, and *otsA* increased at later stages, suggesting improved carbon resource management and stress resilience. The soybean gene *Glyma17g07330* showed variable expression, reflecting dynamic host–microbe interactions.

Structural analysis using transmission electron microscopy revealed that nodules from S141-treated plants contained densely packed bacteroids with abundant poly-β-hydroxybutyrate (PHB) granules [74]. These granules may serve as energy reserves, supporting bacterial viability and prolonging proper function in nodules. In contrast, nodules from single inoculation treatments showed signs of senescence, highlighting the sustaining role of S141.

Field trials further confirmed the physiological benefits of S141. Soybean plants coinoculated with S141 and *Bradyrhizobium* strains produced more nodules, exhibited higher nitrogen fixation rates, and achieved seed yield increases ranging from 9.7% to 43.6% compared to controls [74]. These improvements enhanced nodulation, extended nodule activity and nutrient assimilation.

## 3. *B. velezensis* S141 Genome

The genome of S141 was sequenced, generating a draft assembly of approximately 3.9 megabases with a GC content of 46.4% [75]. The annotation revealed 3853 protein-coding genes, along with 86 tRNA genes and 10 rRNA operons. Phylogenetic analysis confirmed that S141 belongs to the *B. velezensis* species, which is closely related to other PGPR strains such as strains FZB42 and QST713 [76]. However, S141 exhibits distinct genomic features that may underlie its enhanced performance in soybean rhizospheres.

One of the most notable findings is the presence of genes encoding β-glucosidases capable of hydrolysing isoflavone glycosides into aglycones. This enzymatic activity is significant because aglycones are more bioavailable and biologically active than their glycoside counterparts, potentially facilitating improved plant–microbe signalling and root colonisation for *Bradyrhizobium* species to form nodules [77]. Such a trait is either absent or less pronounced in other *B. velezensis* strains [76,78], suggesting that S141 may have evolved specific adaptations to the soybean host environment. As described later, attempts were made to verify this discovery experimentally [79].

In addition to β-glucosidase genes, the genome of S141 contains multiple gene clusters associated with PGPR functions [75]. These include genes involved in the biosynthesis of antimicrobial compounds such as bacillomycin, surfactin, and fengycin, which contribute to biocontrol activity against soil-borne pathogens. Genes related to siderophore production, phosphate solubilisation, and phytohormone modulation, e.g., IAA and cytokinin synthesis, were also identified, supporting the strain’s role in enhancing nutrient uptake and stimulating root development.

## 4. *B. velezensis* S141 Promotes Nodule Development and Nitrogen Fixation Involving IAA and Cytokinins Biosynthesis

The effects of coinoculating *B. velezensis* S141 with various *Bradyrhizobium* strains on nodulation, nitrogen fixation, and soybean growth parameters were also investigated [80]. The results demonstrated significant synergistic effects, showing that S141 enhances the symbiotic efficiency of *Bradyrhizobium* at both phenotypic and molecular levels.

Genome analysis of S141 suggested it possesses several plant growth-promoting traits, including IAA and cytokinin production, phosphate solubilisation, and siderophore production [75]. These traits were confirmed in vitro [75] and supported their potential role in promoting early root development and enhancing microbial interactions in the rhizosphere. Three *Bradyrhizobium* strains were used for co-inoculation: *B. diazoefficiens* USDA110, *B. elkanii* SUTN9-2, and *B. elkanii* SUTN1-12 [80]. These strains were selected based on their established nitrogen-fixing capacities and compatibility with local soybean cultivars in Thailand.

Greenhouse experiments were then conducted to evaluate the impact of single and co-inoculations with *Bradyrhizobium* strains on soybean growth [80]. The co-inoculation of S141 and *B. diazoefficiens* USDA110 yielded the most significant results. This combination resulted in a substantial increase in nodule number—up to 1.7 times greater—along with increased nodule dry weight and total plant biomass, compared to the single inoculation with USDA110 alone.

Nitrogenase activity, measured using the acetylene reduction assay (ARA), was significantly enhanced in co-inoculated plants [80]. For example, the S141 plus USDA110 treatment showed a 1.8-fold increase in ARA activity compared with USDA110 alone. Co-inoculations with the other *Bradyrhizobium* strains also produced improvements, though to a lesser degree. These findings suggest that S141 enhances the nitrogen-fixing efficiency of at least the tested *Bradyrhizobium* strains.

More importantly, disrupting the gene involved in IAA biosynthesis in S141 was found to decrease the formation of large nodules by USDA110 [80]. This result indicates that IAA biosynthesis in S141 impacts soybean growth promotion, supported by efficient nodulation. Disrupting genes related to cytokinin biosynthesis in S141 also resulted in a reduction in extra-large nodules, suggesting that these genes may influence nodule size [80]. However, it is still possible that other substances secreted by S141, aside from IAA and cytokinins, also contribute to nodule formation.

## 5. *B. velezensis* S141 May Process Flavonoids

The enzymatic activities of S141 were also investigated, with particular attention to its ability to hydrolyse soybean isoflavone glycosides—daidzin and genistin—into their corresponding aglycones, daidzein and genistein [79]. These aglycones are critical in legume–rhizobium symbiosis, serving as signals that induce *nod* gene expression in rhizobia [77]. The study further aimed to assess whether S141 possesses a distinctive hydrolytic capacity compared with other *Bacillus* strains and to elucidate the genetic basis of this activity.

S141 exhibited significantly higher hydrolysis of daidzin and genistin than other tested strains, including *B. subtilis* 168 and *B. velezensis* FZB42 [79]. Although kinetic parameters were not reported, the relative efficiency suggests that S141 may play a particularly active role in modifying rhizosphere signalling through isoflavone biotransformation. This elevated activity leads to a specialised enzymatic system not shared by reference strains. Genomic analysis revealed that S141 harbours homologues of four β-glucosidase genes identified in *B. subtilis*: *bglA*, *bglC*, *bglH*, and *gmuD* [75]. Among these, *bglC* is known to account for most isoflavone glycoside hydrolysis in *B. subtilis* [79]. However, the much stronger activity observed in S141 suggests that either its *bglC* homologue is more efficient or that additional, yet unidentified, β-glucosidase genes may also be present [79].

The biological relevance of this enzymatic activity lies in its potential to enhance soybean nodulation; however, this study did not include gene knockout experiments, so the precise contribution of each gene in S141 remains unresolved. Previous studies have shown that co-inoculation of S141 with *B. diazoefficiens* increases both nodulation and nitrogen fixation [74,80]. Hydrolysis of isoflavone glycosides by S141 may contribute to this effect by elevating the availability of daidzein and genistein in the rhizosphere, thereby strengthening the signalling required for effective symbiosis [7]. This represents a distinct PGPR mechanism in which the bacterium modifies plant-derived signalling molecules rather than producing its own.

While the findings are compelling, the study has limitations. It lacks direct genetic evidence linking specific β-glucosidase genes to the observed hydrolysis and does not quantify aglycone concentrations in planta. Furthermore, as the concentration of daidzein and genistein after inoculation is unknown, a causal relationship has not been fully demonstrated. Further research, utilising targeted gene disruption, enzyme purification, and rhizosphere metabolite profiling, will be essential to validate and extend these results.

## 6. *B. velezensis* S141 Promotes Soybean Root Growth Under Drought Stress

*B. velezensis* S141 has recently been investigated for its effects on soybean root growth under drought stress [81]. The study investigated whether S141 alone could promote root development when water availability is limited. Under controlled drought conditions, soybean seedlings treated solely with S141 showed a significant increase in root dry weight relative to untreated controls, demonstrating that S141 enhances root biomass under limited water supply [81]. This finding suggests that S141’s beneficial effects on roots extend beyond symbiotic enhancement; they also promote root growth independently of co-inoculation. However, it remains necessary to verify whether the increase in biomass observed under drought conditions correlates with improved physiological function.

S141 may promote root growth under drought stress via its IAA biosynthesis [80]. However, even though IAA biosynthesis in S141 was disrupted, there was no change in the root growth [81]. This finding suggests that the mechanism promoting root growth under drought conditions may not rely on IAA. Therefore, further experimental approaches are needed to identify alternative signalling molecules, such as peptide-based or volatile organic compounds, that could substitute for IAA in this process.

The study also examined whether S141 activates known drought-responsive plant pathways—specifically osmotic or oxidative stress defences [81]. Soybean plants under drought, irrespective of S141 treatment, displayed typical stress responses. However, S141 did not alter any of these responses, suggesting that its root-growth promotion under drought is not mediated through canonical stress pathways. Although the precise biochemical or molecular mechanisms remain unresolved, the authors hypothesise that S141 secretes unidentified compounds that selectively stimulate soybean root growth under water deficit. Such substances may differ from known phytohormones or stress signals, opening avenues for the discovery of novel bacterial metabolites or signalling molecules underlying PGPR-mediated drought resilience.

## 7. Comparison of *B. velezensis* S141 with Other Bacillus Strains with Agricultural Applications

To verify the specificity of *B. velezensis* S141, we compared it with other *Bacillus* species known for their PGPR functions and agricultural applications. Table 1 summarises the differences among these species in terms of host plant specificity, growth promotion mechanisms, disease suppression, community control, formulation properties, and colonisation abilities.

The PGPR function of S141 is rooted in its host-specific metabolism, directly linking it to the core of symbiosis. It can convert soybean isoflavone glycosides into aglycones via β-glucosidase, thereby enhancing nitrogen fixation through symbiosis with *Bradyrhizobium* [79]. The metabolism specialisation intervening in host phenology distinguishes it from the nutrient acquisition and hormone regulation typical of other PGPR strains. It also produces IAA, which might be involved in enhanced nodulation [80]. Furthermore, its additional function provides resilience to uncertain environments, as it demonstrates the possible drought tolerance (root growth) even under non-symbiotic conditions with *Bradyrhizobium* [81]. Although variations in inoculation and water availability are common in crop fields, S141 has these independent functional axes, providing redundancy and making its effects less likely to be disrupted. This redundancy offers a level of reliability that is hard to achieve with single-function PGPR strains.

Meanwhile, *B. velezensis* FZB42 performs complementary remote effects and community control. It is known to induce root system plasticity from distant locations by secreting volatile organic compounds, thereby redesigning root architecture via the auxin pathway [82]. This function could complement the root growth function in S141, producing a synergistic effect that promotes the formation of the initial root system during drought and nutrient acquisition. Another key characteristic of FZB42 is its ability to control the soybean pathogen *Phytophthora sojae* through the production of bacillicin [83]. When combined with S141, it could maintain enhanced symbiosis under high-disease-pressure field conditions, contributing to yield stabilisation. Furthermore, biofilm dispersion of FZB42 is regulated by c-di-GMP and the *ccdC* genes [84,85], enabling balanced design of colonisation and spread, providing practical insights into the spatiotemporal control of functional expression in the rhizosphere.

*B. amyloliquefaciens* NBRC15535 and the commercial strain D747 broadly express classical PGPR functions, including phosphate solubilisation and production of IAA, siderophores, and antimicrobial substances, and they possess extensive experience in formulation and carrier compatibility [86,87]. In designing synergistic effects within composite formulations, including S141 and FZB42, *B. amyloliquefaciens* is likely to be positioned as a stable benchmark.

*B. toyonensis* BCT-7112T has been extensively studied in relation to safety assessment, immune response enhancement, and applications in livestock and poultry [88,89,90,91,92]. However, its PGPR function remains unconfirmed. Standard indicators such as IAA secretion, phosphate solubilisation, and siderophore production, along with colonisation and biofilm formation, would allow for more detailed comparisons. Additionally, given its history of practical application, it could serve as either a negative or positive control for social implementation in research to validate the efficacy of S141.

These strains exhibit shared core functions characteristic of PGPR *Bacillus*, as each strain possesses capabilities such as IAA secretion, siderophore production, phosphate solubilisation, antimicrobial substance generation, and sporulation [89,90]. Additionally, they demonstrate high applicability, suitability for formulation, storage stability, and adaptability for seed treatment and rhizosphere application, which facilitates easy field introduction [93,94,95,96]. Conversely, focusing on differences, the host-specific metabolism of S141 is markedly more pronounced than that of the others. It also promotes root growth during drought conditions. In contrast, the long-term effects of FZB42, including disease suppression and colonisation control, are features not present in S141. Furthermore, its ability to modulate biofilm enables spatial control of functional expression in the rhizosphere. *B. amyloliquefaciens* possesses commercial reliability, not yet demonstrated by S141. *B. toyonensis* holds value as an exploration target with substantial safety data, serving as a comparison candidate that can clarify the advantages of S141. Combining S141 with FZB42 or *B. amyloliquefaciens* might present an opportunity for an integrated design that includes initial root system formation, symbiotic maturation, and reduced disease pressure. This strategy could make the two goals of reducing fertilisers and stabilising yields within cropping systems much more attainable.

Compared to FZB42, which has been extensively studied, including through genome analysis [97], S141 displays distinct features in several putative genes related to IAA production [75]. These genes include *ipdC*, encoding indole-3-pyruvate decarboxylase for IAA synthesis from indole-3-pyruvate; *dhaS*, encoding indole-3-acetaldehyde dehydrogenase; *iaaH*, encoding indole-3-acetoamide hydrolase for IAA synthesis from indole-3-acetoamide; and *yhcX*, encoding a nitrilase for IAA synthesis from indole-3-acetonitrile. Additionally, *ysnE*, predicted to encode IAA transacetyltransferase involved in the tryptophan-independent IAA biosynthetic pathway, was identified. These results indicate that S141 may play a significant role in auxin production. Analysis of gene clusters for secondary metabolite production using antiSMASH 8.0 [98] (Table 2) shows that S141 could produce all secondary metabolites found in FZB42. Therefore, these metabolites are unlikely to explain the differences in their PGPR functions.

## 8. Discussion

*B. velezensis* S141 was originally isolated from a soybean field in Thailand and has shown promising effects as a PGPR when combined with *Bradyrhizobium* species [74,80]. The core concept is that co-inoculation of PGPR with nitrogen-fixing bacteria enhances plant growth more effectively than either alone. Previous studies demonstrated that PGPR can promote root development, increase flavonoid secretion, and stimulate nodulation [75,81,82,83]. In particular, S141 significantly improved nodulation, nitrogen fixation, and seed yield when co-inoculated with *B. diazoefficiens* USDA110 [74,80]. The beneficial effects appear to arise from multiple mechanisms, including improved root colonisation, greater energy storage in nodules, and delayed senescence. The dual inoculation strategy is consistent with earlier research, showing that mixed microbial communities are often more effective than single-strain inoculants [74,80].

Genomic analysis of S141 supports these findings, revealing a diverse set of genes related to plant-beneficial traits, such as antimicrobial compound biosynthesis, hormone regulation, and nutrient solubilisation [75]. These genomic features help explain its strong performance in promoting plant growth and root symbiosis. Compared with other *B. velezensis* strains, S141 appears particularly well-adapted to interact with soybean roots and rhizobia, making it a valuable model for understanding microbial cooperation in the rhizosphere. S141 may carry β-glucosidase genes that convert soybean isoflavone glycosides into aglycones, enhancing root colonisation and plant signalling [79]. This function is either absent or less active in other strains, including FZB42 and QST713, which rely more on antimicrobial compounds and auxin production [76,99,100,101].

Greenhouse experiments further confirmed the synergistic effects of S141 when co-inoculated with various *Bradyrhizobium* strains [80]. The most pronounced effects were observed with USDA110, resulting in increased nodulation, higher nitrogenase activity, and greater plant biomass. Similar results were obtained among different *Bradyrhizobium* strains, suggesting that the advantage of S141 may be effective not only for USDA110 but also for symbiotic nitrogen fixation by other strains. In vitro assays also confirmed S141’s ability to produce plant growth-promoting substances, including IAA and cytokinins, solubilise phosphate, and release siderophores, all of which may contribute to improved plant nutrition and root development.

An intriguing aspect of S141’s activity is its ability to hydrolyse soybean-derived isoflavone glycosides into their active aglycone forms, which are essential for initiating *nod* gene expression in rhizobia [50,73,79]. This mechanism differs from the more commonly recognised PGPR traits and suggests that S141 strengthens chemical communication between the plant and rhizobia. Its hydrolytic capacity was greater than that of related strains, and genome analysis identified several β-glucosidase genes that may contribute to this activity [79]. However, the study did not directly connect these genes to enzyme function, and further experiments are required to confirm their roles. Nevertheless, this represents a novel way in which PGPR can enhance symbiosis—by modifying plant-derived signalling compounds in the rhizosphere.

Recent findings also demonstrate that S141 promotes root growth in soybeans under drought stress, independent of co-inoculation with rhizobia [81]. Under limited water availability, S141-treated plants developed significantly larger root systems, enhancing their potential for water and nutrient uptake. Unexpectedly, this effect was not dependent on IAA, as disruption of its IAA biosynthesis did not diminish the benefit [81]. Moreover, S141 did not alter the typical drought stress-response pathways of the plants [81]. These results suggest that the bacterium may secrete novel and unidentified compounds that selectively stimulate root growth under stress. This points to a previously unrecognised mode of plant–microbe interaction operating outside known hormone- or stress-response pathways and highlights exciting directions for future research into drought-resilient agriculture.

**Figure 1:**
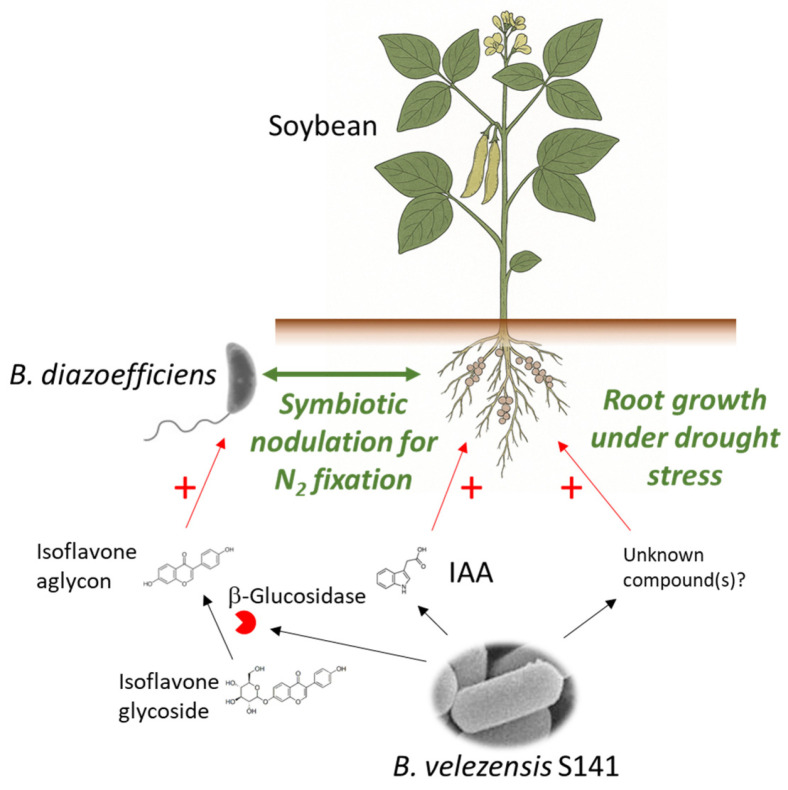
Enhancing soybean growth with *B. velezensis* S141. *B. velezensis* S141 stimulates soybean growth by producing IAA and β-glucosidase to enhance symbiotic nodulation with *B. diazoefficiens* for improved nitrogen fixation and by promoting root growth under drought stress. A + (plus) indicates a promoting effect.

*B. velezensis* S141 can be a versatile and multifunctional PGPR that enhances soybean growth through several complementary mechanisms (Figure 1). From improving root architecture and nutrient uptake to modifying rhizosphere signalling and promoting symbiotic nitrogen fixation, S141 emerges as a promising microbial partner for sustainable crop production systems. Further research into its molecular mechanisms, gene functions, and metabolite profiles will be key to realising its full potential for agricultural applications.

## 9. Future Directions

*B. velezensis* S141 has been extensively studied as a PGPR, demonstrating considerable potential for enhancing plant health and productivity. Beyond its well-documented agricultural benefits, recent research highlights a broader spectrum of applications that position S141 as a multifunctional bioresource in both plant and animal systems [74,80,102,103,104,105]. This section discusses the diverse functionalities of S141, drawing from recent literature to underscore its future potential.

The efficacy of S141 as a PGPR is further supported by studies elucidating a complex tripartite synergistic interaction among the bacterium, arbuscular mycorrhizal fungi, and *Lotus japonicus* [102]. This work demonstrated that S141 not only directly stimulates plant growth but also facilitates symbiotic relationships that enhance nutrient acquisition and stress tolerance in host plants. Such interactions suggest that S141 could play a pivotal role in sustainable agriculture by reducing reliance on chemical fertilisers while simultaneously improving plant resilience under diverse environmental conditions, although verification at full scale, long-term effectiveness, and economic feasibility must be demonstrated.

In addition to its plant growth-promoting activity, the antifungal properties of S141 have also been characterised [103]. Its biocontrol activity was investigated against the ascomycete fungus *Cercospora,* which causes leaf spot in mungbean, revealing that S141 employs multiple mechanisms for its antifungal activity, including the production of lipopeptides and other secondary metabolites that inhibit fungal growth and spore germination. This biocontrol capacity is valuable for integrated pest management strategies, offering an environmentally friendly alternative to chemical fungicides and reducing the ecological burden of synthetic agrochemicals.

Recent advances have further expanded the utility of S141 to other economically important crops. For instance, S141 was reported to significantly promote growth in the annual herbaceous flowering plant *Cannabis sativa* through several mechanisms, including enhanced nutrient acquisition, modulation of phytohormone levels, and induction of systemic resistance against pathogens [104]. These findings suggest that S141 can be applied to optimise the cultivation of diverse crop species beyond traditional legumes, underscoring its versatility in modern horticulture and speciality crop production.

Remarkably, the functional spectrum of S41 extends beyond plant systems, as demonstrated by its probiotic effects in aquaculture [105]. Supplementation of shrimp feed with S141 improved growth performance, enhanced immune responses, and increased tolerance to pathogenic challenges. This novel application highlights the potential of S141 as a sustainable alternative to antibiotics and chemical additives in animal husbandry, contributing to healthier livestock production while addressing global concerns about antimicrobial resistance.

Taken together, these studies underscore the multifaceted nature of S141, which integrates plant growth promotion, biocontrol, and probiotic properties into a single microbial resource. Future research should focus on optimising delivery methods and formulations to maximise the efficacy of S141 in diverse agricultural and aquacultural contexts. Further analyses enable the molecular basis of these interactions to be elucidated in greater detail, thereby facilitating the design of microorganisms tailored to specific environmental or industrial applications.

In conclusion, *B. valezensis* S141 emerges as a promising bio-inoculant with broad applications, ranging from improving crop productivity and protection to supporting animal health in aquaculture. The integration of S141 into sustainable agricultural and aquacultural systems has the potential to enhance food security and foster environmental conservation in the face of global challenges.

## Figures and Tables

**Table 1:** Comparison of *B. velezensis* S141 with other PGPR *Bacillus* strains.

Strain	Host Plant Specificity	Growth Promotion Mechanisms	Disease Suppression and Community Control	Formulation Properties and Colonisation Ability
*B. velezensis* S141	Converting soybean isoflavone glycosides to aglycones enhances *Bradyrhizobium* symbiosis	Root elongation under drought conditions via unknown compound(s)	Indirect (improvement of symbiosis and resource acquisition pathways)	High implementability
*B. velezensis* FZB42	Host specificity is limited	Secretes volatile organic compounds to redesign root architecture via the auxin pathway	Suppression of *P. sojae* through bacillicin production and control of biofilm dispersion via c-di-GMP and *ccdC*	Accumulation of knowledge concerning formulation and fixation
*B. amyloliquefaciens* NBRC15535 and D747	General (wide range of crops)	Classical PGPR functions such as IAA production, phosphate solubilisation, and siderophore secretion	Commercial achievements (disease suppression through lipopeptide secretion)	Adaptation to the substrate has been demonstrated
*B. toyoensis* BCT-7112T	Unestablished	Exploring the potential mechanisms	Derived from feed additives with extensive safety data	PGPR function requires verification

**Table 2:** Presence of gene clusters involved in secondary metabolite production in S141 and FZB42.

Strain	Surfactin	Plantazolicin	Macrolactin H	Bacillaene	Fengycin	Difficidin	Bacillothiazol	Bacillibactin	Bacilysin
S141	+	−	+	+	+	+	−	+	+
FZB42	+	+	+	+	+	+	+	+	+

A minus (−) indicates that the relevant synthetic pathway does not exist, while a plus (+) indicates that it does exist.

## Data Availability

This review does not contain any original data and is based entirely on information presented in previously published studies.

## Abbreviations

The following abbreviations are used in this manuscript:

ARA	acetylene reduction assay
CFU	colony-forming units
IAA	indole-3-acetic acid
PAH	polycyclic aromatic hydrocarbon
PGPR	plant growth-promoting rhizosphere bacteria
PHB	poly-β-hydroxybutyrate
